# Patterns of diaphragm function in critically ill patients receiving prolonged mechanical ventilation: a prospective longitudinal study

**DOI:** 10.1186/s13613-016-0179-8

**Published:** 2016-08-05

**Authors:** Alexandre Demoule, Nicolas Molinari, Boris Jung, Hélène Prodanovic, Gerald Chanques, Stefan Matecki, Julien Mayaux, Thomas Similowski, Samir Jaber

**Affiliations:** 1 INSERM, UMRS1158 Neurophysiologie respiratoire expérimentale et clinique, Sorbonne Universités, UPMC Univ Paris 06, Paris, France; 2 Intensive Care Unit and Respiratory Division (Département “R3S”), Groupe Hospitalier Pitié-Salpêtrière Charles Foix, AP-HP, 75013 Paris, France; 3 Department of Medical Information, Hôpital Arnaud de Villeneuve, IMAG U5149, University of Montpellier, Montpellier, France; 4 INSERM U1046, CNRS UMR 9214, Montpellier School of Medicine, University of Montpellier, Montpellier, France; 5 Intensive Care and Anesthesiology Department, Saint Eloi Hospital, Montpellier, France; 6 Physiology and Experimental Medecine, Heart-Muscle UMR CNRS 9214 – INSERM U1046, Montpellier University, Montpellier, France; 7 Service de Pneumologie et Réanimation Médicale, Groupe Hospitalier Pitié-Salpêtrière, 47-83 boulevard de l’Hôpital, 75651 Paris Cedex 13, France

**Keywords:** Diaphragm Respiratory muscles function, Phrenic nerve stimulation, Intensive care unit, Mechanical ventilation, Sepsis, Outcome

## Abstract

**Background:**

In intensive care unit (ICU) patients, diaphragmatic dysfunction (DD) can occur on admission or during the subsequent stay. The respective incidence of these two phenomena has not been previously studied in humans. The study was designed to describe temporal trends in diaphragm function in mechanically ventilated (MV) patients.

**Methods:**

Ancillary study of a prospective, 6-month, observational cohort study conducted in two ICUs. MV patients were studied within 24 h following intubation (day-1) and every 48–72 h thereafter. Diaphragm function was assessed by twitch tracheal pressure (Ptr,stim) in response to bilateral anterior magnetic phrenic nerve stimulation. Diaphragm dysfunction was defined as Ptr,stim < 11 cmH_2_O. Patients who received MV for at least 5 days were retained, and the first and the last measures were analysed.

**Results:**

Forty-three patients were included. Overall, 79 % of patients developed DD at some point during their ICU stay: 23 (53 %) patients presented DD on initiation of mechanical ventilation, 14 (33 %) of whom had persistent DD, while diaphragm function improved in 9 (21 %). Among the remaining 20 (47 %) patients who did not present DD on initiation of MV, 11 (26 %) developed DD during the ICU stay, while 9 (21 %) did not. Mortality was higher in patients with DD either on initiation of mechanical ventilation or during the subsequent ICU stay than in those who never developed DD (35 vs. 0 %, *p* = 0.04). Duration of MV was higher in patients with DD on initiation of MV that subsequently persisted than in patients who never exhibited diaphragm dysfunction (18 vs. 5 days, *p* = 0.04). Factors associated with a change in Ptr,stim were: age [linear coefficient regression (Coeff.) −0.097, standard error (SD) 0.047, *p* = 0.046], PaO_2_/FiO_2_ ratio (Coeff. 0.014, SD 0.006, *p* = 0.0211) and the proportion of the time under MV with sedation (per 10 %, Coeff. −5.359, SD 2.451, *p* = 0.035).

**Conclusions:**

DD is observed in a large majority of MV patients ≥5 days at some point of their ICU stay. Various patterns of DD are observed, including DD on initiation of mechanical ventilation and ICU-acquired DD.

*Trial registration* clinicaltrials.gov Identifier # NCT00786526

## Background

 Diaphragm function is a major determinant of weaning from mechanical ventilation in intensive care unit (ICU) patients and influences the duration of mechanical ventilation [1]. It has become a major concern in ICU patients and the subject of an increasing number of reports [2].

To date, two major patterns of diaphragm dysfunction have been described in ICU patients. First, the diaphragm, like all organs, can be involved in the shock-related generalized organ failure observed in many patients on admission to the ICU [3]. This occurs in 64 % of patients, is determined by sepsis and the severity of the disease and is associated with higher mortality [3]. Second, diaphragm dysfunction in critically ill patients can occur during the ICU stay in patients without prior diaphragm dysfunction [4, 5]. It can be a consequence of ICU-acquired neuromuscular disorders [6]. It can also be a negative consequence of mechanical ventilation per se, which is associated with a time-dependent decrease of diaphragm strength called ventilator-induced diaphragm dysfunction (VIDD) [7].

The relative distribution of these patterns has not yet been described, since previous reports have focused on only one of these two patterns [3, 5].

The present study addresses this issue by means of longitudinal measurement of tracheal twitch pressure (Ptr,sim) following magnetic stimulation of the phrenic nerve in intubated and mechanically ventilated patients. Ptr,stim was first measured during the first 24 h following intubation and initiation of mechanical ventilation and then every 48–72 h in patients receiving mechanical ventilation for more than 5 days. The study was designed to describe temporal trends in diaphragm function, identify putative clinical factors associated with diaphragm dysfunction and describe the subsequent impact of these changes on the patient’s outcome.

## Patients and methods

The study was conducted over a 6-month period (1 December 2008 to 1 July 2009) in two intensive care units: A 10-bed medical intensive care unit (ICU) (Groupe Hospitalier Pitié-Salpêtrière, Paris) and a 16-bed medical and surgical ICU (Hôpital Saint-Eloi, Montpellier). The study was approved by the “*Comité de Protection des Personnes Sud*-*Méditerrannée II*”, Montpellier, France. All patients or their relatives provided written informed consent to participate. Data from this cohort have been presented in previously published studies [3, 5]. This study was registered in the US National Institutes of Health clinical trials registry (clinicaltrials.gov NCT00786526).

### Patients

Patients were eligible for inclusion in the study within the 24 h following intubation and initiation of mechanical ventilation. Exclusion criteria were an expected duration of mechanical ventilation less than 48 h, contraindications to magnetic stimulation of the phrenic nerves (cardiac pacemaker or implanted defibrillator, cervical implants), use of neuromuscular blocking agents within the 24 h preceding the first diaphragm function assessment (with the exception of succinylcholine used during rapid-sequence induction of anaesthesia for intubation), pre-existing neuromuscular disorders, cervical spine injury, factors possibly interfering with tracheal pressure measurements in response to phrenic stimulation (multiple functioning chest drains, severe chronic obstructive pulmonary disease). Finally, age less than 18 years, known pregnancy, and a decision to withhold life-sustaining treatment also constituted exclusion criteria.

### Diaphragm assessment

Diaphragm performance was assessed in terms of changes in endotracheal tube pressure induced by bilateral phrenic nerve stimulation during airway occlusion (Ptr,stim). The first Ptr,stim measurement was performed within 24 h of intubation, and, whenever possible, these measurements were repeated every 48–72 h until extubation or death.

Phrenic nerve stimulation was performed by bilateral anterior magnetic stimulation [8]. Briefly, two figure-of-eight coils connected to a pair of Magstim^®^ 200 stimulators (The Magstim Company, Dyfed, UK) were positioned immediately posterior to the sternocleidomastoid muscles at the level of the cricoid cartilage. Stimulations were delivered at the maximum intensity allowed by the stimulator. The patients were studied in a standardized semirecumbent position as follows: end-expiratory pressure was set to zero, and the patient was allowed to exhale during an end-expiratory pause until expiratory airflow reached zero (relaxed equilibrium volume of the respiratory system). The endotracheal tube was then occluded, and bilateral anterolateral magnetic stimulation was performed. The absence of active respiratory efforts in response to stimulation was determined by checking the stability of the airway pressure signal. Measurements were repeated at least three times and were performed by the two same operators in each centre. Stimulations were always performed by the same two operators in each centre. Ptr,stim was defined as the amplitude of the negative pressure wave following stimulation, determined from baseline to peak. It was measured at the proximal end of the endotracheal tube, using a linear differential pressure transducer (MP45 ± 100 cmH_2_O, Validyne, Northridge, CA, USA). The pressure signal was sampled and digitized at 128 Hz (MP30, Biopac Systems, Santa Barbara, CA, USA or Powerlab, AD Instruments, Bella Vista, Australia) for subsequent data analysis.

### Clinical data collection

Demographic data, severity scores, organ dysfunction-related variables, presence of sepsis [9] and blood gas data were recorded prospectively on ICU admission, at day 1. Administration of mechanical ventilation, sedation and catecholamines was recorded each day. The durations of mechanical ventilation, ICU stay and hospital stay were also recorded, as were decisions to perform tracheotomy, ICU mortality and hospital mortality.

### Statistical analysis

Because the present study was designed to investigate temporal trends in diaphragm function during mechanical ventilation, only patients who received mechanical ventilation for at least 5 days were retained and the first and the last measures were considered in the analysis. Diaphragm dysfunction was defined as Ptr,stim < 11 cmH_2_O [3, 10, 11].

Patients were further qualified according to four patterns according to the presence or absence of diaphragm dysfunction on initiation of MV and during the subsequent ICU stay:

1. “Persistent Dysfunction” defined patients with diaphragm dysfunction on initiation of MV (Ptr,stim < 11 cmH_2_O) and persistent diaphragm dysfunction during the ICU stay (Ptr,stim remained < 11 cmH_2_O),
2. “Improving Dysfunction” defined patients with diaphragm dysfunction on initiation of MV (Ptr,stim < 11 cmH_2_O), but in whom diaphragm function improved during the ICU stay (Ptr,stim increased to ≥ 11 cmH_2_O),
3. “Acquired Dysfunction” defined patients without diaphragm dysfunction on initiation of MV (Ptr,stim ≥ 11 cmH_2_O), but who acquired diaphragm dysfunction (Ptr,stim decreased to < 11 cmH_2_O) during the ICU stay,
4. “No Dysfunction” defined patients without diaphragm dysfunction on initiation of MV (Ptr,stim ≥ 11 cmH_2_O) and in whom diaphragm function remained normal throughout the ICU stay (Ptr,stim remained ≥ 11 cmH_2_O).

Factors associated with these patterns of diaphragm function as well as factors associated with changes in diaphragm function were assessed. These factors were: age, gender, body mass index, SAPS II, type of admission, SOFA, sepsis, blood gases, blood lactate, sedation, amines and controlled mechanical ventilation (as opposed to partial modes). The relationship between potential factor of diaphragm dysfunction and the four predefined patterns of diaphragm dysfunction was evaluated in a univariate model (one-way ANOVA or Kruskal–Wallis test for continuous variables depending on distribution; Chi-square test or Fisher’s exact test for categorical variables depending on size). In addition, in order to analyse the level of Ptr,stim as a continuous endpoint over time, linear mixed effects models were performed.

The impact of time trend of Ptr,stim on ICU and hospital mortality, tracheostomy rate, duration of mechanical ventilation and length of stay was also assessed in a univariate model.

For all final comparisons, a *p* value ≤0.05 was considered statistically significant without any correction for multiple comparisons. Statistical analysis was performed with R software (version R.2.13.2). The median and interquartile range (IQR) are reported for continuous variables, and absolute and relative frequencies are reported for categorical variables.

## Results

### Study population

Eighty-five patients were enrolled during the study period [3]: Forty-three of them were both mechanically ventilated ≥5 days and had ≥3 Ptr,stim measurements. The mean age was 61 (54–77) years, and the mean SAPS 2 score was 53 (44–65).

### Prevalence and factors associated with changes in diaphragm function in the ICU

**Fig. 1:**
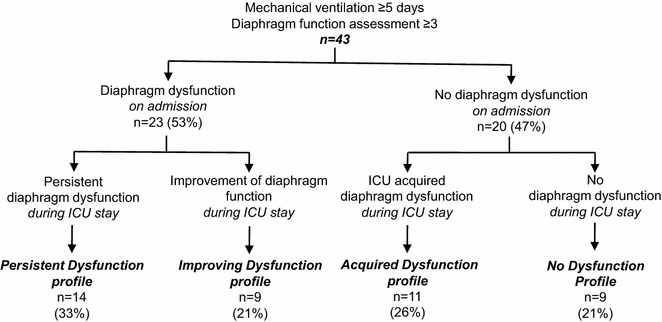
Study flow chart. *ICU* intensive care unit

Figure 1 displays the distribution of patients according to the four predefined patterns. Twenty-three patients presented diaphragm dysfunction on admission, 14 (33 %) of whom had persistent diaphragm dysfunction (“Persistent Dysfunction” pattern) and 9 (21 %) had improvement of diaphragm function (“Improving Dysfunction” pattern). Among the remaining 20 patients who did not present diaphragm dysfunction on admission, 11 (26 %) subsequently developed diaphragm dysfunction (“Acquired Dysfunction” pattern) and 9 (21 %) did not present any diaphragm dysfunction during the ICU stay (“No Dysfunction” pattern). Overall, 34 (79 %) patients developed DD at some point of their ICU stay.

**Fig. 2:**
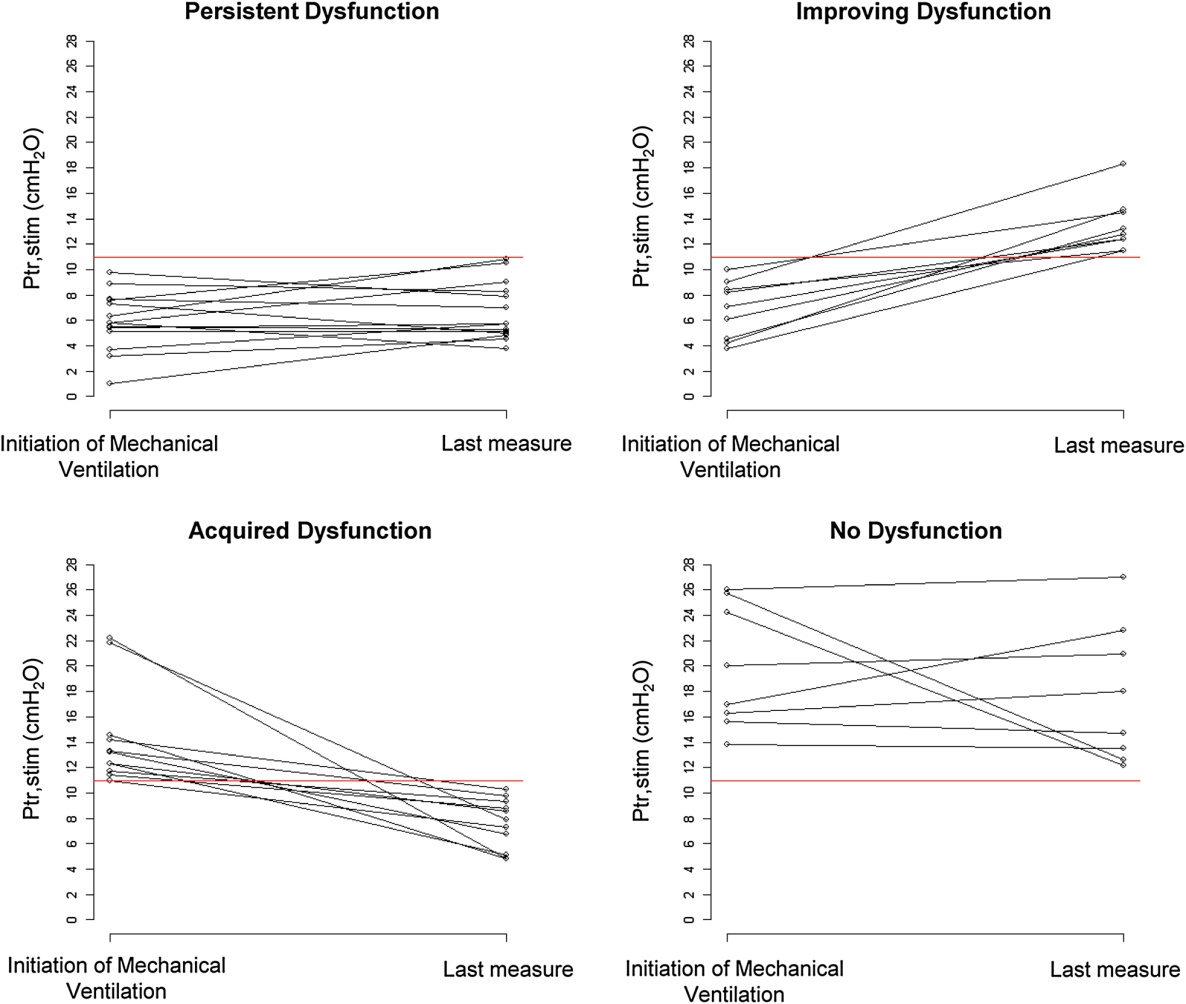
Individual endotracheal tube pressure induced by bilateral phrenic nerve stimulation (Ptr,stim) on admission and during the subsequent intensive care unit stay in the four groups defined by the four patterns

Figure 2 depicts the value of Ptr,stim on initiation of MV and during ICU. In patients with a “Persistent Dysfunction” pattern, Ptr,stim was 5.8 (5.1–7.6) cmH_2_O on initiation of MV and did not change during the ICU stay [4.6 (3.8–5.7) cmH_2_O after 7.5 (5.0–13.0) days, *p* = 0.313]. In patients with an “Improving Dysfunction” pattern, Ptr,Stim increased from 7.1 (4.5–8.4) cmH_2_O on initiation of MV to 12.8 (12.4–14.5) cmH_2_O after 7.0 (6.0–13.0) days (*p* = 0.004). Ptr,stim on ICU admission was not different between patients with a “Persistent Dysfunction” pattern and those with an “Improving Dysfunction” pattern (*p* = 0.395). In patients with an “Acquired Dysfunction” pattern, Ptr,stim deceased from 13.2 (11.7–14.6) cmH_2_O on initiation of MV to 7.9 (5.1–9.3) cmH_2_O after 7.0 (4.0–11.0) days (*p* = 0.001). Finally, in patients with a “No Dysfunction” pattern, Ptr,stim was 20.0 (16.3–25.7) cmH_2_O on initiation of MV and did not decrease [20.9 (15.8–25.1) cmH_2_O after 4.0 (3.0–7.0) days, *p* = 0.839]. Ptr,stim on admission was significantly lower in patients with an “Acquired Dysfunction” pattern than in those with a “No Dysfunction” pattern (*p* = 0.005).

Univariate analysis showed that, among patients with diaphragm dysfunction on initiation of MV, patients with an “improving dysfunction” pattern tended to have a lower body mass index (Table 1). In patients without diaphragm dysfunction on ICU admission, no risk factor was associated with subsequent acquisition of diaphragm dysfunction (Table 2).

**Table 1:** Characteristics of the study population on intensive care unit admission

	Diaphragm dysfunction on admission *n* = 23		No diaphragm dysfunction on admission *n* = 20		*p*
	Persistent dysfunction *n* = 14	Improving dysfunction *n* = 9	ICU-acquired dysfunction *n* = 11	No dysfunction *n* = 9	
Age (years)	75 (54–78)	58 (48–70)	62 (54–72)	59 (53–79)	0.508
Gender (M)	8 (57)	3 (33)	7 (63)	6 (66)	0.515
BMI (kg/m^2^)	27.1 (23.8–29.1)	22.4 (19.4–24.7)	25.1 (23.8–30.2)	24.4 (23.8–26.1)	0.095
SAPS II	55 (48–70)	61 (32–71)	45 (44–57)	49 (47–53)	0.510
Type of admission: medical [*n* (%)]	10 (71)	8 (89)	8 (73)	7 (78)	0.859
Indication for MV					0.069
Shock [*n* (%)]	1 (7)	1 (13)	0	0	
Coma [*n* (%)]	3 (21)	0	5 (45)	6 (67)	
ARF [*n* (%)]	10 (71)	8 (88)	6 (55)	3 (38)	

Continuous variables are expressed as median (interquartile range), and categorical data are expressed as number (%)

*ICU* intensive care unit, *BMI* body mass index, *SAPS* simplified acute physiology score, *MV* mechanical ventilation

**Table 2:** Characteristics of the study population at first diaphragm assessment and during intensive care unit stay

	Diaphragm dysfunction on admission *n* = 23		No diaphragm dysfunction on admission *n* = 20		*p*
	Persistent dysfunction *n* = 14	Improving dysfunction *n* = 9	ICU-acquired dysfunction *n* = 11	No dysfunction *n* = 9	
*At first diaphragm assessment*					
SOFA	9 (8–10)	9 (5–11)	8 (7–8)	6 (4–10)	0.573
Sepsis [*n* (%)]	11 (79)	7 (78)	6 (55)	3 (33)	0.121
PaO_2_/FiO_2_ (mmHg)	211 (141–302)	208 (143–281)	202 (120–295)	314 (120–460)	0.593
PaCO_2_ (mmHg)	38 (33–45)	36 (31–37)	33 (31–38)	35 (31–39)	0.635
pH	7.41 (7.31–7.46)	7.41 (7.33–7.47)	7.39 (7.33–7.49)	7.45 (7.31–7.49)	0.854
Blood lactate (mmol/L)	2.5 (1.3–3.2)	1.5 (1.2–2.9)	2.3 (1.7–3.4)	1.9 (1.2–3.00)	0.626
*During ICU stay*					
Sedation (% of ICU stay)	54 (30–75)	67 (25–71)	50 (33–73)	33 (13–43)	0.302
Amines (% of ICU stay)	100 (0–100)	100 (0–100)	6 (0–100)	0 (0–71)	0.189
Controlled MV (% of ICU stay)	64 (43–80)	71 (25–83)	50 (33–75)	33 (25–43)	0.610

Continuous variables are expressed as median (interquartile range), and categorical data are expressed as number (%)

*ICU* intensive care unit, *SOFA* sequential organ failure assessment, *MV* mechanical ventilation

When Ptr,stim was assessed as a continuous variable, factors associated with a change in Ptr,stim were: age [linear coefficient regression (Coeff.) −0.097, standard error (SD) 0.047, *p* = 0.046], PaO_2_/FiO_2_ ratio (Coeff. 0.014, SD 0.006, *p* = 0.0211) and the proportion of the time under MV with sedation (per 10 %, Coeff. −5.359, SD 2.451, *p* = 0.035).

### Clinical outcomes

Table 3 displays the major clinical outcomes. ICU mortality was higher in patients with a “Persistent Dysfunction” pattern than in those with a “No Dysfunction” pattern (*p* = 0.03). Moreover, mortality was higher in patients with diaphragm dysfunction either on initiation of MV or during the subsequent ICU stay than in those in whom diaphragm dysfunction was never observed (35 vs. 0 %, *p* = 0.04).

**Table 3:** Main clinical outcomes

	Diaphragm dysfunction on admission *n* = 23		No diaphragm dysfunction on admission *n* = 20		*p*
	Persistent dysfunction *n* = 14	Improving dysfunction *n* = 9	ICU-acquired dysfunction *n* = 11	No dysfunction *n* = 9	
Duration of MV (days)	18 (15–21)	10 (7–20)	11 (6–18)	5 (4–7)	0.012
Tracheotomy, [*n* (%)]	3 (23)	2 (25)	1 (9)	1 (13)	0.799
ICU mortality [*n* (%)]	7 (54)	1 (13)	4 (37)	0	0.037
Hospital mortality [*n* (%)]	8 (62)	1 (13)	4 (37)	2 (25)	0.139
ICU LOS (days)	21 (18–27)	13 (10–24)	15 (9–30)	8 (7–14)	0.212
Hospital LOS (days)	21 (18–44)	27 (12–47)	17 (9–72)	18 (13–24)	0.832

Continuous variables are expressed as median (interquartile range), and categorical data are expressed as number (%)

*ICU* intensive care unit, *MV* mechanical ventilation, *LOS* length of stay

Duration of mechanical ventilation was higher in patients with a “Persistent Dysfunction” pattern than in those with a “No Dysfunction” pattern (*p* = 0.04). Among patients with diaphragm dysfunction on initiation of MV, the duration of mechanical ventilation tended to be higher in those with a “Persistent Dysfunction” pattern than in those with an “Improving Dysfunction” pattern (*p* = 0.072). Among patients without diaphragm dysfunction at the time of intubation, the duration of mechanical ventilation tended to be higher in those with an “Acquired Dysfunction” pattern than in those with a “No Dysfunction” pattern (*p* = 0.064).

## Discussion

This study shows that mechanically ventilated ICU patients can exhibit various patterns of diaphragm function, defined as the capacity of the diaphragm to produce a negative intrathoracic pressure in response to phrenic nerve stimulation. Overall, 79 % of patients developed diaphragm dysfunction at some point of their ICU stay.

Two major patterns of diaphragm dysfunction have been described in humans. The first of these two patterns is diaphragm dysfunction on initiation of MV. Like all striated muscles, the diaphragm can be involved in the shock-related organ failure observed in many patients on admission to the ICU [3], in which sepsis plays a major role [12]. This phenomenon has been clearly established for the heart [13]. It also involves limb muscles, as suggested by electromyographic and histopathological studies conducted soon after ICU admission. These studies have shown that more than 50 % of patients with severe sepsis exhibit signs of neuromuscular damage [14]. Diaphragm dysfunction on admission was observed in 64 % of patients in our first cohort [3] and in 53 % of patients in the present study, which is ancillary to the first cohort. The difference between these two rates can be explained by the fact that only patients mechanically ventilated for at least 5 days were included in the present study. Because diaphragm dysfunction on admission is associated with higher mortality [3], some patients died before day 5 and were therefore not included in the analysis. The second pattern of diaphragm dysfunction described in humans is ICU-acquired diaphragm dysfunction in mechanically ventilated patients [4, 5, 15], which is partly a consequence of mechanical ventilation, known as ventilator-induced diaphragm dysfunction (VIDD). It results from striated muscle disuse atrophy [15] and injury [16]. In addition, diaphragm strength is also sensitive to ICU-acquired neuromuscular disorders [6] and hypercatabolism and corticosteroid use [17] that are frequently observed in the ICU.

To the best of our knowledge, this is the first study to report the relative incidence of these two phenomena: 53 % of patients presented diaphragm dysfunction on initiation of MV and 26 % presented no diaphragm dysfunction on admission, but subsequently developed diaphragm dysfunction. We also show that these two phenomena may be interrelated. For instance, 33 % of the patients who presented diaphragm dysfunction on ICU initiation of MV never recovered, as VIDD may have contributed to prevent any form of recovery in these patients. It is noteworthy that mortality tended to be higher in the subgroup of patients in whom diaphragm function did not recover, suggesting that the combination of these two phenomena may worsen the prognosis. Twenty-one per cent of patients had a diaphragm dysfunction on initiation of MV and subsequently recovered, suggesting that even severe diaphragm dysfunction on admission can be reversed. This is consistent with echocardiographic and physiological findings in patients with sepsis-associated myocardial dysfunction, which show reversibility over a period of 7–10 days [18]. Finally, 21 % of patients never exhibited diaphragm dysfunction, either on initiation of MV or during ICU stay. Although this study was not designed to assess causality, it is noteworthy that the proportion of the time under mechanical ventilation spent on a controlled mode of ventilation as opposed to a partial mode of ventilatory assistance was associated with the decrease of Ptr,stim. This finding supports the contribution of controlled mechanical ventilation and subsequent diaphragm rest to the pathogenesis of ICU-acquired diaphragm dysfunction.

Our data are original since this is to the best of our knowledge the first study to provide longitudinal data on diaphragm function on initiation of MV and during the subsequent ICU stay. Among the few studies that describe diaphragm function in the ICU with Ptr,stim, none has longitudinally described patterns of diaphragm function in a cohort of similar size. The two the largest series studied patients either upon initiation of mechanical ventilation or at various time points, but the time trend in diaphragm function was not described [3, 19]. Two studies have reported variation of diaphragm function during ICU stay, but on much smaller samples [4, 5]. Finally, in a recent report, diaphragm function was studied in 40 patients with ICU acquired diaphragm weakness, but only at the time of extubation [20].

One of the strengths of this study is that diaphragm strength was tested by bilateral phrenic nerve stimulation, a gold standard method that provides a specific measurement of the capacity of the diaphragm to generate an inspiratory pressure [21]. Many reports of ultrasound or CT-scan evaluation of diaphragm morphology in ICU patients have been published [22–24]. However, these techniques do not constitute the gold standard for assessment of diaphragm function and few data are available to establish a correlation between ultrasound measurements and Ptr,stim [20], which remains the gold standard for assessment of diaphragm function in ICU patients. Another strength is that the study was conducted in two centres and the study population is representative of standard ICU recruitment.

Our study presents several limitations. Diaphragm function was assessed by measuring tracheal pressure (Ptr,stim) without the use of oesophageal and gastric probes to measure transdiaphragmatic pressure, which would have been more precise, but also more invasive, and much less practical in a large cohort. We cannot rule out that other factors aside from intrinsic diaphragm dysfunction may have contributed to changes in Ptr,stim. This would be the case of a decrease in positive end-expiratory pressure and subsequent improvement in diaphragm length–tension relations. However, patients with severe chronic obstructive pulmonary disease were not included. There is a clear lack of power due to the limited sample size. We were therefore unable to identify any independent risk factors for improvement or worsening of diaphragm function. Finally, our choice of a diaphragm dysfunction cut-off of 11 cmH_2_O may be questionable. However, this choice was based on published reports [10, 11], was validated in a population of patients briefly anaesthetized and mechanically ventilated for elective surgical procedures and was subsequently used in a previous report [3].

## Conclusion

 A large majority of patients mechanically ventilated for at least 5 days exhibit diaphragm dysfunction at some point during their ICU stay. Diaphragm dysfunction presents many different patterns in these patients, comprising diaphragm dysfunction on admission and ICU-acquired diaphragm dysfunction. Larger studies are needed to determine the risk factors for diaphragm function changes and the clinical consequences of these changes in mechanically ventilated ICU patients.
